# Effects of Linguistic Distance on Second Language Brain Activations in Bilinguals: An Exploratory Coordinate-Based Meta-Analysis

**DOI:** 10.3389/fnhum.2021.744489

**Published:** 2022-01-06

**Authors:** Elisa Cargnelutti, Barbara Tomasino, Franco Fabbro

**Affiliations:** ^1^Dipartimento/Unità Operativa Pasian di Prato, Scientific Institute, IRCCS E. Medea, Udine, Italy; ^2^Cognitive Neuroscience Laboratory, Department of Languages, Literature, Communication, Education, and Society, University of Udine, Udine, Italy; ^3^Institute of Mechanical Intelligence, Scuola Superiore Sant’Anna, Pisa, Italy

**Keywords:** bilingualism, Ginger-ALE meta-analysis, fMRI, linguistic distance, language families, age of appropriation (AoA)

## Abstract

In this quantitative meta-analysis, we used the activation likelihood estimation (ALE) approach to address the effects of linguistic distance between first (L1) and second (L2) languages on language-related brain activations. In particular, we investigated how L2-related networks may change in response to linguistic distance from L1. Thus, we examined L2 brain activations in two groups of participants with English as L2 and either (i) a European language (European group, *n* = 13 studies) or (ii) Chinese (Chinese group, *n* = 18 studies) as L1. We further explored the modulatory effect of age of appropriation (AoA) and proficiency of L2. We found that, irrespective of L1-L2 distance—and to an extent—irrespective of L2 proficiency, L2 recruits brain areas supporting higher-order cognitive functions (e.g., cognitive control), although with group-specific differences (e.g., the insula region in the European group and the frontal cortex in the Chinese group). The Chinese group also selectively activated the parietal lobe, but this did not occur in the subgroup with high L2 proficiency. These preliminary results highlight the relevance of linguistic distance and call for future research to generalize findings to other language pairs and shed further light on the interaction between linguistic distance, AoA, and proficiency of L2.

## Introduction

The majority of the world population is bilingual. They speak two or more languages (Grosjean, 2010), which may even belong to different language families, namely, groups of languages or dialects with a common progenitor sharing a certain degree of similarity at lexical, phonological, and morphosyntactic levels (Rowe and Levine, 2018). The following four main macrolinguistic families have been identified: (i) African and Southwest-Asian family, (ii) European and North-Asian family (including the Indo-European family), (iii) East-Asian, Southeast-Asian, and Australian family (including the Sino-Tibetan family), and (iv) American family (Wichmann et al., 2012). Language families were identified by comparing languages for linguistic features as well as analyzing genetic data and corresponding evolutionary trees of the populations speaking those languages (Cavalli-Sforza et al., 1992; Henn et al., 2012; Reich, 2018).

The Indo-European language family is one of the most commonly spoken languages worldwide. It includes ancient languages such as Greek, Latin, and Sanskrit and many modern European languages (e.g., English, French, German, Italian, Modern Greek, Spanish, and Russian), but also languages spoken in the Indian subcontinent (e.g., Hindi) and in the Iranian region (e.g., Fārsí) (Beekes, 2011). English is spoken by 900 million individuals, Hindi by 570 million, and Spanish by 400 million. The other most commonly spoken language family is Sino-Tibetan, with Mandarin being spoken by approximately one billion individuals (Corballis, 2017).

Languages differ structurally to a certain degree and are thus characterized by different degrees of linguistic distance. It is not easy to determine such distance as languages may be similar in some respects and differ in other respects. For instance, Chinese and English are similar in some aspects of syntax as both have a subject-verb-object order, but they markedly differ in writing and phonology.

To establish a quantitative index for linguistic distance, several methods have been developed, which compare languages at lexical, phonological, and morphosyntactic levels (Spielman et al., 1974). Most commonly, the lexical similarity between two languages is examined. Several automatic systems have been developed to compare a limited number of words (up to 100) which are considered fundamental (Holman et al., 2008a). Accordingly, linguistic distance is defined as the overall number of additions, deletions, or substitutions of symbols (e.g., letters) necessary to change a given word in a given language into the corresponding word in the target language (Levenshtein distance). Recently, this method was used to automatically determine the linguistic distance between languages belonging to different families (Holman et al., 2008b; Serva and Petroni, 2008; Wichmann et al., 2010). Other approaches focused on qualitative and quantitative differences in phonemes (Atkinson, 2011) or on syntactic differences (Comrie, 1989).

Nevertheless, since such comparison may be a reductive criterion, other scholars (Hart-Gonzalez and Lindemann, 1993; Chiswick and Miller, 2005) operationalized linguistic distance in terms of the difficult people normally encounter when learning a specific language. Accordingly, Hart-Gonzalez and Lindemann (1993) monitored the average performance scores achieved by English-speaking individuals on a newly learned language following a training period. Based on the proven assumption that learning a new language is easier when this is structurally close (vs. distant) to the native language (Crystal, 1987), performance scores were taken as an inverse index of linguistic distance (Chiswick and Miller, 2005), with scores ranging from 1.00 to 3.00, indicating languages that are hardest and easiest to learn by native English speakers.

With regard to brain representation of languages in bilingual individuals, different languages are associated with specific cerebral microcircuits, involving both overlapping and distinct cortical areas (Fabbro, 1999; Paradis, 2004). Generally, second language (L2) is represented more extensively than native language (L1) and involves brain regions subserving cognitive control, in particular, in bilinguals who have learned their L2 later and/or had low L2 proficiency/exposure, as shown in previous meta-analyses on both healthy bilinguals (Sebastian et al., 2012; Liu and Cao, 2016; Cargnelutti et al., 2019; Sulpizio et al., 2020) and bilinguals with aphasia (Kuzmina et al., 2019). The age at which appropriation of L2 (i.e., Age of Appropriation, AoA)^1^ (Paradis, 1994, 2009; Ullman, 2001, 2005, 2006) occurred and the level of proficiency associated with L2 (Sebastian et al., 2012; Cargnelutti et al., 2019) are the two main factors shaping brain representation of languages in bilinguals.

Linguistic distance may be another potentially important aspect contributing to the different L1 vs. L2 brain representations. Several crosslinguistic studies compared the functional networks associated with processing Chinese or Japanese vs. English showing that differences, for instance in phonology or writing, translate into the recruitment of different brain circuits (Bolger et al., 2005; Tan et al., 2005). Studies focused on bilinguals enable to understand how the bilingual brain deals with different processing demands associated with each language.

Nevertheless, this factor has not been systematically studied yet, in particular, in neuroimaging studies on bilinguals, in which quantification of the linguistic distance between the languages spoken by tested bilinguals is not normally assessed. Although this quantification is more a matter of linguistics than neuroscience, a few interesting results can be found in neuroimaging studies too.

For instance, Jeong et al. (2007a) investigated the functional brain networks associated with each language in native Korean trilinguals with English as L2 and Japanese as a third known language (L3). Both these languages were learned late (mean AoA 12.3 and 20.6, respectively). Proficiency in L2 and L3 was comparable. All three languages belong to different language families, although Korean is more similar to Japanese than English (e.g., they have similar syntactic structures and are left-branching, agglutinative languages). The results showed comparable functional activation during auditory sentence comprehension between Korean (L1) and Japanese (L3), whereas English (L2) determined additional activations in both cortical (e.g., pars opercularis of left inferior frontal gyrus and right superior temporal cortex) and subcortical (i.e., right cerebellar hemisphere) regions. This study clearly shows that the brain functional networks associated with each language are also shaped, besides AoA and proficiency, by structural differences between languages.

A recent meta-analysis addressed the role that the writing system has on L2 brain representation (Liu and Cao, 2016). The authors observed that, when L2 is orthographically shallower (i.e., more transparent) than L1, the primary sensorimotor cortex and phonological processing areas are primarily activated, reflecting regularity in grapheme-phoneme conversion. On the contrary, when L2 is orthographically deeper (i.e., opaquer), higher-order frontal regions are recruited to meet additional cognitive demands. These findings suggest that bilinguals may rely on different processes when using L2; these processes can be the same as, or differ from, those associated with L1, depending on how much the latter successfully meet L2 demands, therefore, depending also on linguistic distance.

### This Study

Considering that an increasing percentage of the population worldwide masters even structurally distant languages, this study aimed to investigate how L1-L2 linguistic distance may have an effect on L2 brain representation. We carried out a quantitative meta-analysis to investigate the role of this factor, although the linguistic distance was not directly assessed or quantified in the studies we selected.

In particular, we focused on studies with bilinguals speaking English (i.e., an Indo-European language) as L2 and either a European language with Latin script or Chinese (i.e., a Sino-Tibetan language) as L1. These specific language groups were chosen for two reasons as follows: the overall marked linguistic distance between English and Chinese (Chiswick and Miller, 2005; Wichmann et al., 2012) and the fact that there is an adequate number of neuroimaging studies involving these language pairs. As detailed below, we limited our choice to European languages with Latin script, which are phylogenetically close to English and have a close common progenitor, as shown by several language trees (Serva and Petroni, 2008; Chang et al., 2015). Furthermore, as reported in Chiswick and Miller (2005), English appeared to be structurally much closer to the selected Indo-European languages (with a score of 2.25–2.50) than to Chinese (with a score of 1.00). In other words, we aimed to inspect whether linguistic distance as derived from this index can actually translate into differential English-L2 functional activations.

Second, we controlled for the effect of L2 AoA and proficiency, given that both these factors were identified by prior meta-analyses as fundamental influencers of the L2 language network in healthy bilinguals (Sebastian et al., 2012; Liu and Cao, 2016; Cargnelutti et al., 2019; Sulpizio et al., 2020) and bilinguals with aphasia (Kuzmina et al., 2019). Specifically, given that late AoA and low proficiency are likely to pose greater L2 demands and therefore translate into additional functional activations, we investigated whether these factors could further modulate the effect of linguistic distance on L2 brain representation.

We provided an almost exploratory description because only a few studies to date have explored these specific bilingual patterns. However, we believed that investigating how L2-related network changes in response to L1-L2 linguistic distance are a relevant topic of growing interest. With our current preliminary findings, we also aimed to prompt further research in the field using different language pairs and a better conceptualization of the linguistic distance concept.

## Methods

### Paper Selection

We performed a keyword search (i.e., “fMRI” or “functional MRI,” “PET,” “bilingual*,” “multilingual*”) in databases such as PubMed/MedLine, Scopus, and Scholar to extract papers published in the 1995–2019 period and focused on language representation in the bilingual brain. This sample was complemented by papers identified from inspection of the reference lists of the identified papers. We selected peer-reviewed papers written in English, a process that could lead to a publication bias. However, coordinate-based meta-analyses look for spatial convergence between reported coordinates and thus they differ from effect-size meta-analyses, which quantify the effect size, and are instead prone to bias. Current meta-analyses are less prone to region- and task-dependent biases and less affected by the exclusion of unpublished data (Fox et al., 1998; Rottschy et al., 2012). To limit potential sources of bias, we excluded findings related to *a priori* selected ROIs and chose to include only those from whole-brain analyses.

**FIGURE 1:**
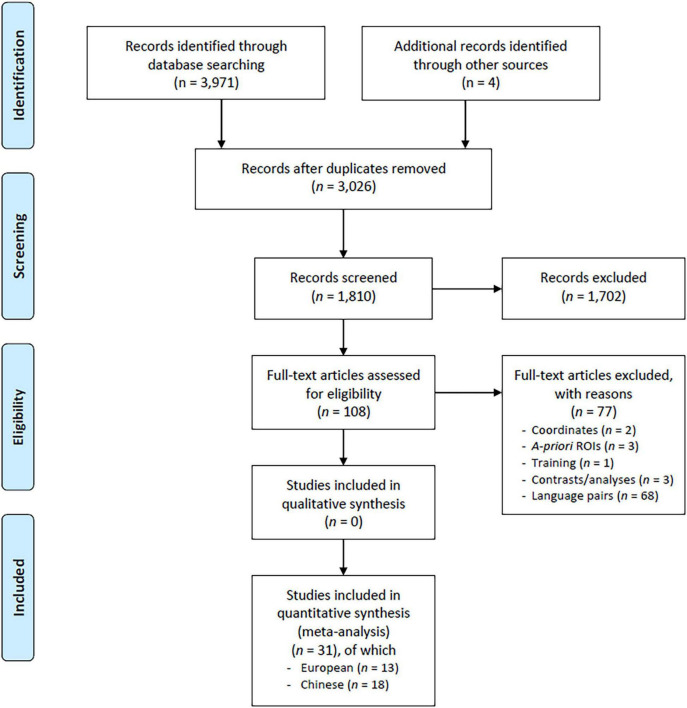
PRISMA flowchart. Schematic representation of the paper search and selection processes.

The paper selection procedure is shown in Figure 1 (PRISMA flow chart, Moher et al., 2009). We included only studies performed on healthy adult individuals (aged 18–60 years) addressing the main language structural domains (i.e., lexical semantics, phonology/articulation, and morphosyntax, see Sebastian et al., 2012; Liu and Cao, 2016). To reduce data variability, we excluded studies investigating non-purely linguistic tasks (e.g., affective/emotional components of language, numbers, and mathematics) and tasks that were specific to bilinguals (e.g., translation/interpretation, switching, and language control). The latter tap additional brain processes as both languages are simultaneously activated during these tasks and, therefore, deserve a specific meta-analysis (Luk et al., 2011). We also excluded studies assessing language performance after either a learning/training period or some kind of manipulation in the degree of exposure to a given language.

The paper selection consisted of several steps, which were guided by the literature and the language pairs spoken by bilinguals in the eligible papers. Paper inclusion was ultimately determined by the languages addressed in the candidate papers and by how much they were represented in the literature. In the first step, we scrutinized all studies with bilinguals having an Indo-European language as L2 and either another Indo-European language or one of the languages spoken in Eastern Asia as L1 (following language classification in Wichmann et al., 2012 and measures of language distance in Chiswick and Miller, 2005).

This first selection resulted in 108 potentially candidate papers, which were then accurately read to possibly exclude papers due to: (i) absent or incomplete (not full 3D) coordinates, only coordinate ranges (i.e., no single coordinate corresponding to the peak activation in a given functional cluster), coordinates reported only for a single subject (*n* = 2); (ii) coordinates from *a priori* selected ROIs (*n* = 3); (iii) analyses with contrasts not being informative (e.g., they did not differentiate between different languages or between bilinguals and monolinguals) or that were too specific or related to a very low-level of linguistic processing, such as passive viewing of single letters (*n* = 3); (iv) assessment after language training (*n* = 1).

A sample of 99 papers survived this scrutiny as they all met the criteria for our meta-analysis. Then, we focused on the available language pairs. We observed that East-Asian languages considered as L1 included Chinese, Korean, and Japanese, which belong to three different language families (i.e., Sino-Tibetan, Koreanic, and Japonic, respectively). In the large majority of these studies, L2 was English. This led us to choose this language as a common denominator between the two groups. Concerning L1, given that only the studies with Chinese-native speakers had the number of experiments (*n* > 15) sufficient to perform robust meta-analyses, we restricted the analyses to this language. In the group with an Indo-European language as L1, we chose languages with less linguistic distance from English (Chiswick and Miller, 2005; Serva and Petroni, 2008; Chang et al., 2015), namely, alphabetic European languages with Latin script (as previously described). Then, Dutch, French, German, Italian, Portuguese, and Spanish were included (and Bengali, Hindi, Macedonian, and Fārsí excluded). The resulting sample consisted of 31 studies: 13 studies with a European language as L1 and 18 studies with Chinese as L1. Hereafter, we named the two groups as European group and Chinese group, respectively. The full list of papers is reported in Table 1.

**TABLE 1:** List of all the studies included in the meta-analysis.

	Authors	*N*	L1	L2	AoA	Participants’ age	L2 proficiency	Technique	Type of task	Type of contrast
**(i)**										
	^a,b^ Berken et al. (2015)	16 Simultaneous b; 13 sequential b	French	English	From birth, for simultaneous b; 13.9 (5), for sequential b	Simultaneous b: 23.3(3.1); sequential b: 25.2(4.2)	High	fMRI	Sentence reading	L2>L1
	^a,b^ Buchweitz et al. (2012)	11	Portuguese	English	13.08 (3.1; range: 10–22)	29.9 (5.74; range: 20–40)	High	fMRI	Inner speech production from different semantic category items	L2>Baseline
	^a^ Golestani et al. (2006)	10	French	English	Range: 10–12	Range: 20–28	Varying (overall, moderately fluent)	fMRI	Sentence generation from single words	L2>Baseline
	^c^ Hernandez et al. (2015)	20	Spanish	English	3.95 (2.17)	21.55 (2.14; range: 18–26)	Higher than L1	fMRI	Word reading	L2>Baseline
	^b^ Kovelman et al. (2008)	11	Spanish	English	From birth or at 4–5 (at school)	19 (range: 18–22)	High (>80% accuracy in screening task)	fMRI	Semantic judgment on sentences with classic or unusual word order	L2>L1
	^b,c^ Jamal et al. (2012)	12	Spanish	English	3.79 (2.21)	22.67 (range: 18–29)	Comparable to L1	fMRI	Word reading while focusing on letter font	L2>Baseline; L2>L1
	^c^ Meschyan and Hernandez (2006)	12	Spanish	English	4.33 (1.16)	22.3 (1.35; range: 20–25)	Higher than L1	fMRI	Word reading	L2>Baseline; L2>L1
	^a, c^ Nosarti et al. (2009)	17	Italian	English	16 (range: 11–21)	31	Self-ratings: 75.8(12.1)/100	fMRI	Regular- and irregular-word reading	L2>Baseline; L2>L1
	^a,b^ Perani et al. (1998)	9 High-proficiency b; 9 low-proficiency b	Italian	English	After 10	High-proficiency b: 19–50 (range); low-proficiency b: 21–32 (range)	High for one group, low for the other	PET	Story listening (comprehension)	L2>Baseline
	^a^ Perani et al. (1996)	9	Italian	English	After 7	Range: 21–32	Moderate	PET	Story listening (comprehension)	L2>Baseline
	^a^ Reiterer et al. (2013)	26	German	English	Around 10	28.38 (5.0) and 25.46 (5.0) (two subgroups characterized by a different ability to pronounce an unknown language)	Varying	fMRI	Sentence reading (focus on pronunciation)	L2>Baseline
	^a,b,c^ van Heuven et al. (2008)	12	Dutch	English	Mean around 11	24.1 (range:19–30)	Self-ratings: around 5–6/7	fMRI	Lexical decision	L2: Words>pseudo-words; L2:bilinguals> monolinguals
	^a^ Waldron and Hernandez (2013)	11 early b; 11 late b	Spanish	English	For early b: 3.18 (1.53; range: 1–6); for late b: 11 (3.33; range: 7–17)	Early b: 21.5 (1.7); late b: 24.9 (4.1)	Not explicitly stated	fMRI	Past-tense verb generation	L2>Baseline; L2>baseline, late>early b.
**(ii)**										
	^a^ Cao et al. (2014)	15	Chinese	English	11.7 (range: 9–13)	22.9 (2.3)	Varying	fMRI	Word reading with rhyming judgment	L2>Baseline
	^a^ Cao et al. (2013)	26	Chinese	English	11.7 (range: 9–13)	22 (range: 19–27)	Varying	fMRI	Word reading with rhyming judgment	L2>Baseline
	^c^ Chan et al. (2008)	11	Chinese	English	Range: 3–5	Range: 21–32	Significantly lower than L1	fMRI	Lexical decision	L2>baseline
	^b,c^ Chee et al. (2001)	9	Chinese	English	5 or before	Range: 23–34	High	fMRI	Semantic association between words or characters	L2>Baseline; L2>L1
	^b,c^ Chee et al. (2000)	6	Chinese	English	5 or before	Range: 20–23	High	fMRI	Semantic association between words or characters	L2>Baseline
	^a,b,c^ Ding et al. (2003)	6	Chinese	English	12.17 (range: 11–13)	22.67 (Range: 21–24)	High	fMRI	Word reading either with focus to word font or with semantic categorization	L2>Baseline; L2>L1
	^a^ Feng et al. (2015)	40	Chinese	English	12.2 (after 10)	23.2 (1.5)	Low to intermediate	fMRI	Story reading (comprehension)	L2>L1
	^a,^ Jeong et al. (2007b)	12	Chinese	English	12.1 (1.2)	26 (Range:19–35)	Level 2 in Society for Testing English Proficiency	fMRI	Sentence comprehension	L2>Baseline; L2>L1
	^a,b,c^ Klein et al. (1999)	6	Chinese	English	12.1 (range: 10–14)	29 (Range:19–45)	> 90% Accuracy on four different tasks	PET	Noun-to-verb generation	L2>Baseline
	^a,c^ Li et al. (2013)	15	Chinese	English	10.64 (2.59)	24.44 (3.43)	Moderate	fMRI	Picture naming	L2>L1; L2: Bilinguals> monolinguals
	^a,c^ Liu et al. (2010)	24	Chinese	English	Around 12	21.8 (2.15)	Self-ratings: 5.87/10	fMRI	Picture naming	L2>L1
	^a,c^ Luke et al. (2002)	7	Chinese	English	After 10	Range: 20–31	Varying (self-ratings: 5.86(0.90)/7 for reading, 5.29(1.60)/7 for speaking)	fMRI	Syntactic and semantic plausibility judgment of phrases	L2>Baseline
	^b,c^ Nelson et al. (2009)	11	Chinese	English	N/A	Not specified (but University students)	High	fMRI	Passive viewing of written words and characters	L2>Baseline
	Nichols and Joanisse (2016)	22	Chinese	English	13.82 (7.12; Range 4–30)	22.18 (4.24; range: 18–35)	Accuracy in a proficiency test: 68.28(6.62)/100	fMRI	Judgment on picture-auditory word matching	L2>L1
	^a,c^ Sun et al. (2015)	13	Chinese	English	12	20.3 (range:18–23)	Self-ratings: around 5–6/10	fMRI	Word and character reading	L2>L1; English: Chinese-English b >English-Chinese b
	^a,b^ Tan et al. (2003)	12	Chinese	English	12 On average	Range: 20–39	Moderately high	fMRI	Word reading with rhyming task	L2>Baseline
	Wang et al. (2010)	12	Chinese	English	N/A	Range: 21–26	Not explicitly stated	fMRI	Word reading	L2>Baseline
	^a,c^ Yang et al. (2011)	17	Chinese	English	Around 12	21.78 (range: 19–28)	Self-ratings: around 4–5/7	fMRI	Lexical decision on words and characters	L2>L1

*If not otherwise indicated, values related to the age of appropriation and age of participants correspond to the mean age for the sample (with eventual SD in parentheses).*

*AoA, age of appropriation; L1, first language; L2, second language; N/A, not available. In (i) studies with European L1; in (ii) studies with Chinese as L1.*

*^a^Studies included in the AoA analysis.*

*^b^Studies included in the proficiency analysis.*

*^c^Studies included in the lexical-semantics analysis (reported in Supplementary Results).*

### Statistical Analyses

The analyses were carried out using the GingerALE software (brainmap.org), relying on a coordinate-based activation likelihood estimation (ALE) algorithm, looking for consistency in functional coordinates across reported contrasts (Turkeltaub et al., 2012; Eickhoff et al., 2009; Laird et al., 2009a,b). This algorithm is based on a random-effect approach that accounts for spatial uncertainty by treating the reported foci as centers for 3D Gaussian probability distributions. The provided probability distribution maps, which were weighted on the number of subjects, described the probability for a given focus to be within a given voxel.

We performed the following analyses:

(i) Investigation of the functional brain networks associated with the two languages, and specifically with L2, in both groups and subsequent comparison between the two groups. We were confident in including different language tasks in the same analysis, given that the algorithm, as just described, looks for areas showing a convergence of activation across different experiments and therefore provides only consistently recurring activations. This choice was also motivated by the restricted number of selected studies, which prevented robust analyses for specific linguistic domains, tasks, or stimulus presentation modalities. However, in Supplementary Results, we reported an exploratory analysis on lexical semantics, which was the most frequently represented domain.

(ii) Investigation of the role of AoA. After carrying out the analysis on the whole groups of bilinguals—which we expected to provide the most general and robust functional activations irrespective of influencers –, we identified two groups of early and late bilinguals following the most commonly adopted AoA cutoff (i.e., L2 appropriation either before or after the age of 6 years). We found that studies including early bilinguals (AoA < 6) were limited in number, especially for the Chinese group. For this reason, we restricted the analyses to late bilinguals. We expected the analysis to focus on late bilinguals to better highlight potential differences between the two groups.

(iii) Investigation of the role of L2 proficiency. Assessment of proficiency is sometimes inappropriate for the purpose of systematic analyses. Accordingly, many studies reported either qualitative or quantitative—yet non-objective—proficiency rates (e.g., self-ratings in rating scales). Only a small percentage of studies tested the proficiency level by a comprehensive screening and standardized batteries, even though neither proved an optimal measure of proficiency (Grosjean, 2010). Although conscious of possible biases, we retained only studies declaring a high proficiency or with high self-rating or screening test scores. As long as proficiency was observed to reduce differences in brain activations between L1 and L2 (Sebastian et al., 2012), it was interesting to investigate to what extent these differences could be modulated by linguistic distance.

We performed (i) main effect analyses, which provide results consisting in the functional activations associated with a specific condition (i.e., L2 in the European group and L2 in the Chinese group) and (ii) contrast analysis, which provide conjunction results, namely, the areas activated in both conditions (i.e., in both the European and the Chinese groups), and subtraction results, which reveal specific activations, namely, areas emerging from the direct comparison between the two conditions and being activated in one condition but not in the other (i.e., the functional activations of the European group survived after subtracting activations of the Chinese group and vice versa).

To set thresholds for both main effect and contrast analyses, we followed the software user instructions and reference articles (Eickhoff et al., 2016; Xu et al., 2020), in order to use approaches already observed to be highly reliable. For main effect analyses, we used a *p* < 0.05 permutation-based cluster-level family-wise error (FWE) corrected threshold (1,000 permutations; voxel-wise threshold *p* < 0.001, uncorrected) and a minimum cluster size of 200 mm^3^ (25 voxels). This is a conservative thresholding approach. For contrast analyses, we based on the conservative minimum statistics (Nichols et al., 2005); therefore, only areas that resulted to be significant in the individual analyses were included. We thresholded probability values at *p* < 0.001, with 10,000 *p*-value permutations and minimum cluster size of 80 mm^3^ (10 voxels); however, for the conjunction results, we only retained clusters of at least 120 mm^3^ (15 voxels), in order to avoid incidental overlap between individual ALE maps (Rottschy et al., 2012; Krall et al., 2015).

We transferred the coordinates to Montreal Neurological Institute (MNI) standard space. Coordinates in Talairach and Tournoux (1988) space were converted to the MNI space by icbm_spm2tal transform before running the analyses. Anatomical localization and labeling of resultant clusters of activation were performed using the SPM Anatomy toolbox (Eickhoff et al., 2005), which assigns activations to the most probable cytoarchitectonic area.

## Results

### L2

**FIGURE 2:**
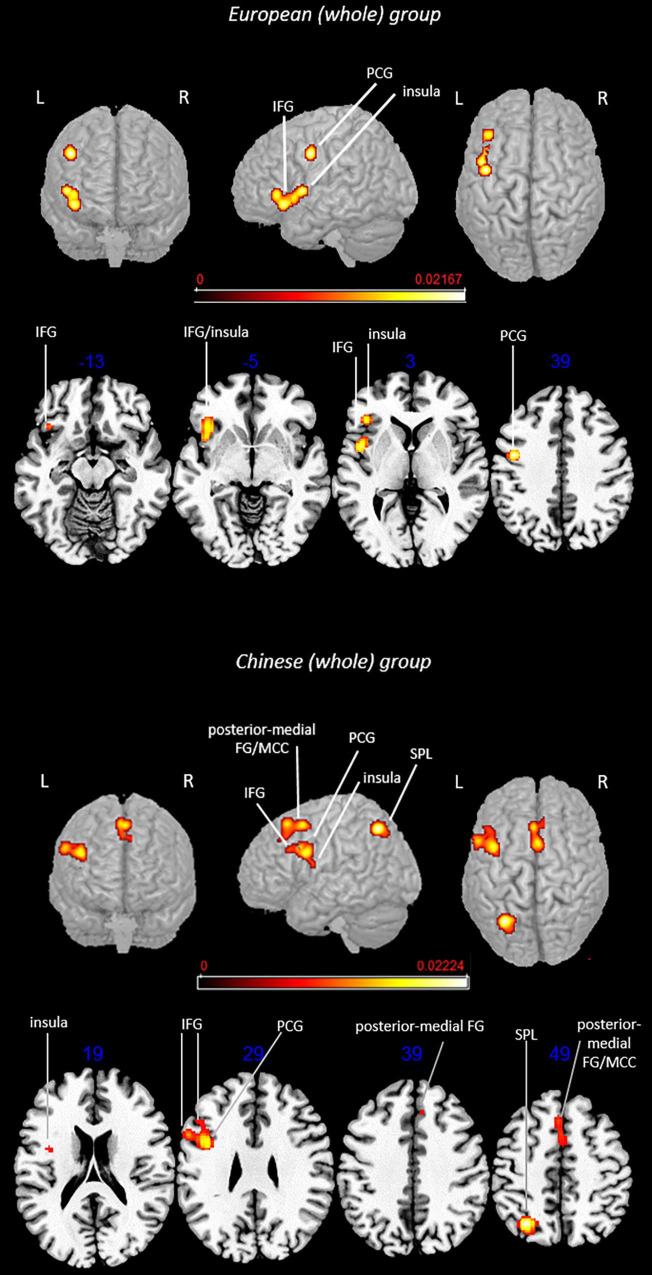
Rendered functional activations associated with L2 (i.e., English) main effects. Rendered anatomical depiction (in neurological convention) of main effect results associated with L2 in the European and Chinese (whole) groups. IFG, inferior frontal gyrus; L, left hemisphere; MCC, mid-cingulate cortex; PCG, precentral gyrus; posterior-medial FG, posterior-medial frontal gyrus; R, right hemisphere; SPL, superior parietal lobule. On axial slices, numbers in blue indicate *z*-coordinates in MNI space. Bars indicate ALE values.

**FIGURE 3:**
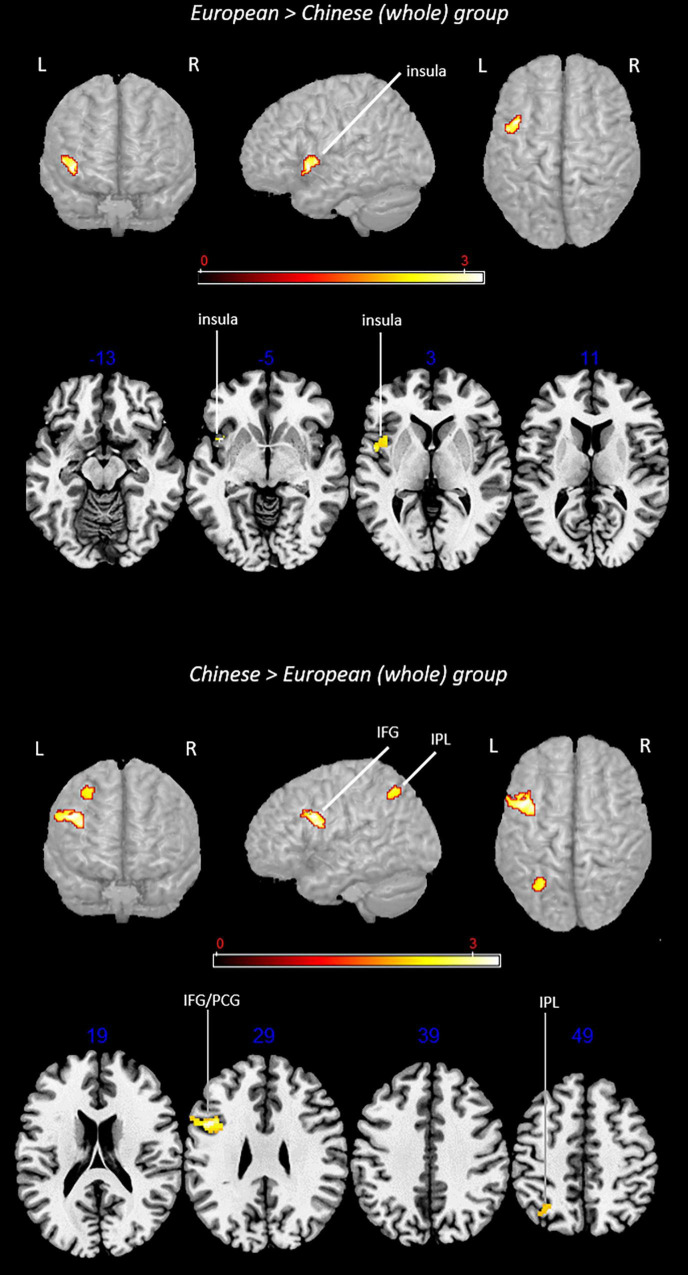
Rendered functional activations associated with L2 (i.e., English) specifically for the European and Chinese groups. Rendered anatomical depiction (in neurological convention) of contrast analysis results specifically associated with L2 in the European and Chinese (whole) groups. IFG, inferior frontal gyrus; IPL, inferior parietal lobule; L, left hemisphere; R, right hemisphere. On axial slices, numbers in blue indicate *z*-coordinates in MNI space. Bars indicate *z*-scores.

Results of both main effect and contrast analyses are reported in Table 2. Figure 2 shows the results from main effect analyses and Figure 3 from contrast analyses.

**TABLE 2:** Results of main effect and contrast activation likelihood estimation (ALE) meta-analyses for L2 (i.e., English) in the European and Chinese (whole) groups.

Cluster (local maxima)		MNI coordinates			Cluster size (voxels)	*z*-score
		x	y	z		
**L2—European group** (30 experiments, 368 subjects, and 265 foci)						
1	L precentral gyrus	–42	–4	38	116	5.15
2	L inferior frontal gyrus	–38	22	–10	439	4.87
	L insula lobe	–46	4	4		
	L inferior frontal gyrus	–40	28	0		
	L insula lobe	–42	12	–2		
**L2—Chinese group** (24 experiments, 320 subjects, and 173 foci)						
1	L superior parietal lobule (area hIP3)	–30	–64	48	271	5.63
2	L inferior frontal gyrus	–40	6	26	436	4.91
	L precentral gyrus	–56	12	32		
	L insula lobe	–38	0	18		
	L inferior frontal gyrus	–44	20	30		
3	R posterior-medial frontal gyrus	2	10	52	324	4.77
	L posterior-medial frontal gyrus	–2	24	54		
	R mid cingulate cortex	6	26	40		
**L2—European group ∩ Chinese group** (54 experiments, 688 subjects, and 438 foci)						
No suprathreshold clusters						
**L2—European group > Chinese group** (54 experiments, 688 subjects, and 438 foci)						
1	L insula	–40	14	–10	97	3.09
**L2—Chinese group > European group** (54 experiments, 688 subjects, and 438 foci)						
1	L inferior parietal lobule (area hIP3)	–34	–62	46	65	2.74
2	L inferior frontal gyrus	–42	6	24	242	3.29

*Anatomical localization, macroanatomic area and, when provided, cytoarchitectonic location (in parentheses) are indicated.*

#### Main Effect: European (Whole) Group

This analysis included 30 experiments, 368 subjects, and 265 foci. L2-associated brain activations emerged in the following regions of the left hemisphere: (i) precentral gyrus, (ii) inferior frontal gyrus (including regions associable with the dorsolateral prefrontal cortex, DLPFC), and (iii) insula.

#### Main Effect: Chinese (Whole) Group

This analysis included 24 experiments, 320 subjects, and 173 foci. L2 activation clusters in the group of bilinguals with Chinese as L1 included left-lateralized activations in the (i) superior parietal lobule (SPL) (area hIP3), (ii) inferior frontal gyrus (including regions associable with the DLPFC), (iii) precentral gyrus, and (iv) insula; activations in both hemispheres were observed for the (iv) posterior-medial frontal gyrus.

Contrast analyses included 54 experiments, 688 subjects, and 438 foci.

#### Conjunction Analysis: European (Whole) Group ∩ Chinese (Whole) Group

The analysis did not provide any suprathreshold clusters.

#### Subtraction Analysis: European (Whole) Group > Chinese (Whole) Group

This analysis showed a specific activation in the (i) left insula.

#### Subtraction Analysis: Chinese (Whole) Group > European (Whole) Group

This contrast provided activations in the left (i) inferior parietal lobule (IPL) (area hIP3) and (ii) inferior frontal gyrus (region including the DLPFC).

### L2 in Late Bilinguals

**FIGURE 4:**
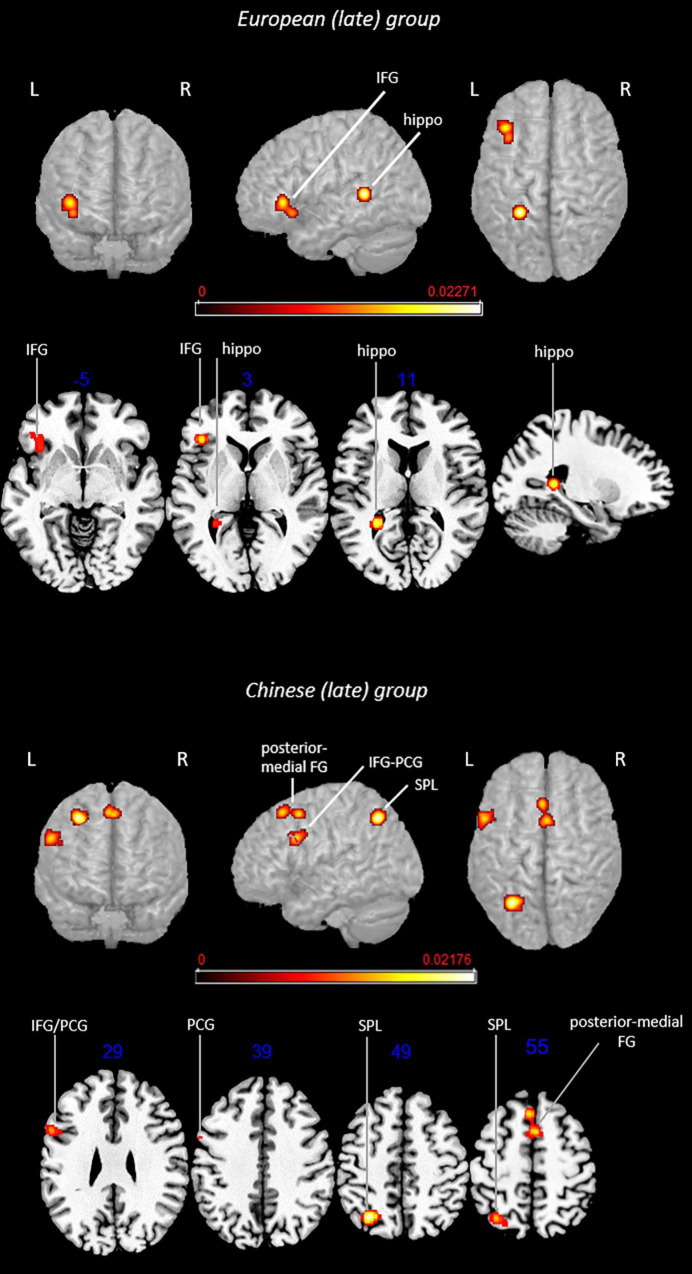
Rendered functional activations associated with L2 (i.e., English) main effects in late bilinguals. Rendered anatomical depiction (in neurological convention) of main effect results associated with late-learned L2 in the European and Chinese groups. hippo, hippocampus; IFG, inferior frontal gyrus; L, left hemisphere; PCG, precentral gyrus; posterior-medial FG, posterior-medial frontal gyrus; R, right hemisphere; SPL, superior parietal lobule. On axial slices, numbers in blue indicate *z*-coordinates in MNI space. Bars indicate ALE values.

**FIGURE 5:**
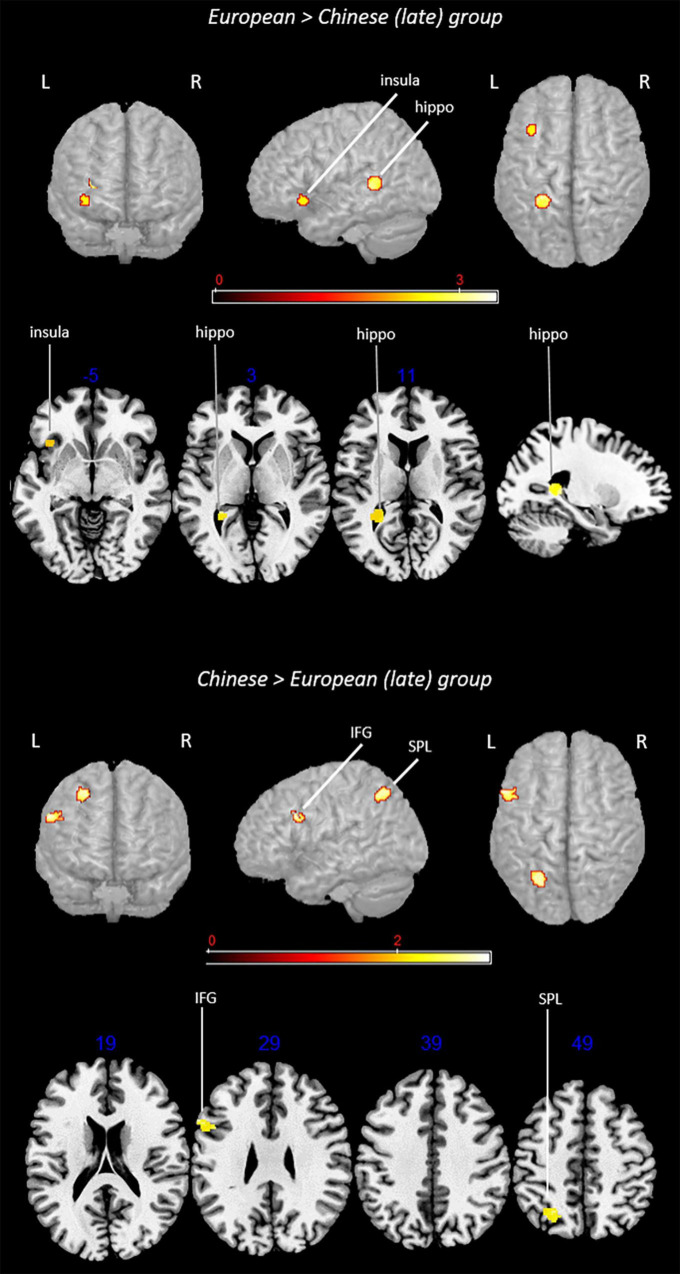
Rendered group-specific functional activations associated with L2 (i.e., English) in late bilinguals. Rendered anatomical depiction (in neurological convention) of contrast analysis results specifically associated with late-learned L2 in the European and Chinese groups. hippo, hippocampus; IFG, inferior frontal gyrus; L, left hemisphere; R, right hemisphere; SPL, superior parietal lobule. On axial slices, numbers in blue indicate *z*-coordinates in MNI space. Bars indicate *z*-scores.

The main effect and contrast analysis results are detailed in Table 3. Figure 4 shows the results from main effect analyses and Figure 5 from contrast analyses.

**TABLE 3:** Results of main effect and contrast ALE meta-analyses for late-learned L2 and proficient L2 (i.e., English) in the European and Chinese groups.

Cluster (local maxima)		MNI coordinates			Cluster size (voxels)	*z*-score
		x	y	z		
**Late-learned L2**						
*European group (16 experiments, 188 subjects, and 135 foci)*						
1	N/A (hippocampus)	–26	–44	8	153	6.01
2.	L inferior frontal gyrus	–40	28	0	223	5.11
	L inferior frontal gyrus	–38	20	–10		
*Chinese group (16 experiments, 230 subjects, and 120 foci)*						
1	L superior parietal lobule (area 7A)	–30	–64	50	241	5.83
2	L precentral gyrus (BA 44)	–56	12	32	177	4.65
3	L posterior-medial frontal gyrus	–2	24	54	187	4.70
	R posterior-medial frontal gyrus	2	10	52		
*European group*∩ *Chinese group (32 experiments, 418 subjects, and 255 foci)*						
No suprathreshold clusters						
*European group* > *Chinese group (32 experiments, 418 subjects, and 255 foci)*						
1	L N/A (hippo)	–30	–48	2	153	3.43
2	L insula	–38	18	–12	49	2.60
*Chinese group* > *European group (32 experiments, 418 subjects, and 255 foci)*						
1	L superior parietal lobule (area hIP3)	–28	–60	44	125	2.74
2	L inferior frontal gyrus	–58	10	26	72	2.97
**Proficient L2**						
*European group (12 experiments, 140 subjects, and 113 foci)*						
1	L superior frontal gyrus	–20	18	56	92	4.75
2	L insula	–38	20	–8	212	4.85
3	R insula	36	26	–6	99	4.73
*Chinese group (11 experiments, 119 subjects, and 54 foci)*						
1	L inferior frontal gyrus	–44	10	28	310	4.46
	L inferior frontal gyrus	–44	20	30		
	L inferior frontal gyrus	–50	18	20		
2	L posterior-medial frontal gyrus	–2	24	54	312	5.50
	L posterior-medial frontal gyrus	0	14	50		
	L posterior-medial frontal gyrus	–4	20	66		
3	L inferior frontal gyrus	–30	32	6	85	4.29
*European group*∩ *Chinese group (33 experiments, 259 subjects, and 167 foci)*						
No suprathreshold clusters						
*European group* > Chinese group (33 experiments, 259 subjects, and 167 foci)						
	L inferior frontal gyrus	–40	18	–14	160	2.31
*Chinese group* > *European group (33 experiments, 259 subjects, and 167 foci)*						
1	L inferior frontal gyrus	–44	8	24	224	2.70
2	L posterior-medial frontal gyrus	–4	16	50	69	2.04

*Anatomical localization, macroanatomic area and, when provided, cytoarchitectonic location (in parentheses) are indicated.*

*^1^We used the term appropriation instead of the traditional term acquisition because—as pointed out by Paradis (2009)—appropriation is more general as it includes both acquisition—which relies on implicit processes mainly characterizing early language appropriation—and learning—which relies on explicit processes mainly characterizing late language appropriation.*

*^2^Parietal activation was not found in L1 main effect analysis (see Supplementary Results), probably because of the strict thresholds we applied and the small number of studies included. Two clusters emerged in SPL (46 voxels, peaking at x = –24, y = –66, z = 40, and 42 voxels, peaking at x = –32, y = –62, z = 56) with more permissive thresholds only. This hypothesis could be confirmed in future by further studies.*

#### Late-Learned L2 in the European Group

This analysis included 16 experiments, 188 subjects, and 135 foci. L2-associated activation clusters were found in the following regions of the left hemisphere: (i) area not matching with any probability map (located in the hippocampus) and (ii) inferior frontal gyrus (including regions associable with the DLPFC).

#### Main Effect: Late-Learned L2 in the Chinese Group

This analysis included 16 experiments, 230 subjects, and 120 foci. L2-associated functional activations included left-lateralized activations in the (i) SPL (area 7A) and (ii) precentral gyrus (BA 44), whereas bilateral activation involved the (iii) posterior-medial frontal gyrus.

Contrast analyses included 32 experiments, 418 subjects, and 255 foci.

#### Conjunction Analysis: Late-Learned L2 in the European Group ∩ Late-Learned L2 in the Chinese Group

The analysis did not provide any suprathreshold clusters.

#### Subtraction Analysis: Late-Learned L2 in the European Group > Late-Learned L2 in the Chinese Group

Direct group comparison showed activations in the left (i) area not matching with any probability map (located in the hippocampus) and (ii) insula.

#### Subtraction Analysis: Late-Learned L2 in the Chinese Group > Late-Learned L2 in the European Group

This contrast provided activations in the left (i) SPL (area hIP3) and (ii) inferior frontal gyrus (region including the DLPFC).

### L2 in Proficient Bilinguals

**FIGURE 6:**
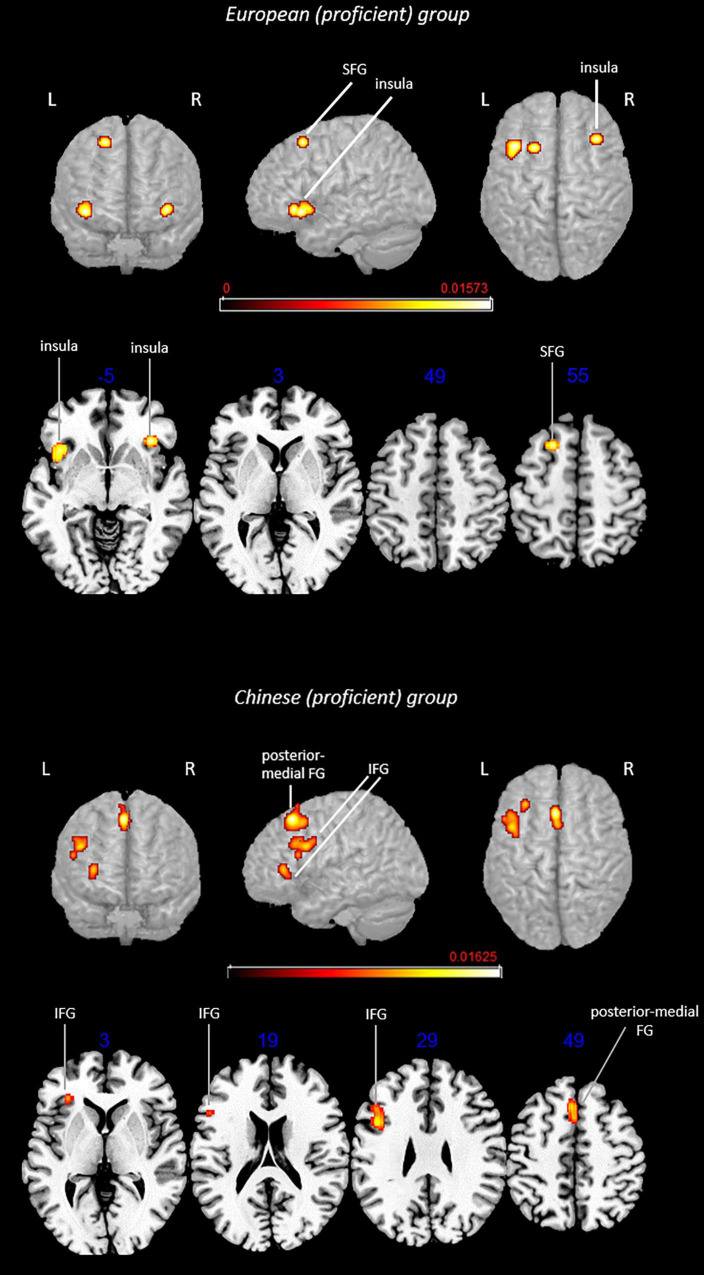
Rendered functional activations associated with L2 (i.e., English) main effects in proficient bilinguals. Rendered anatomical depiction (in neurological convention) of main effect results associated with proficient L2 in the European and Chinese groups. IFG, inferior frontal gyrus; L, left hemisphere; posterior-medial FG, posterior-medial frontal gyrus; R, right hemisphere; SFG, superior frontal gyrus. On axial slices, numbers in blue indicate *z*-coordinates in MNI space. Bars indicate ALE values.

**FIGURE 7:**
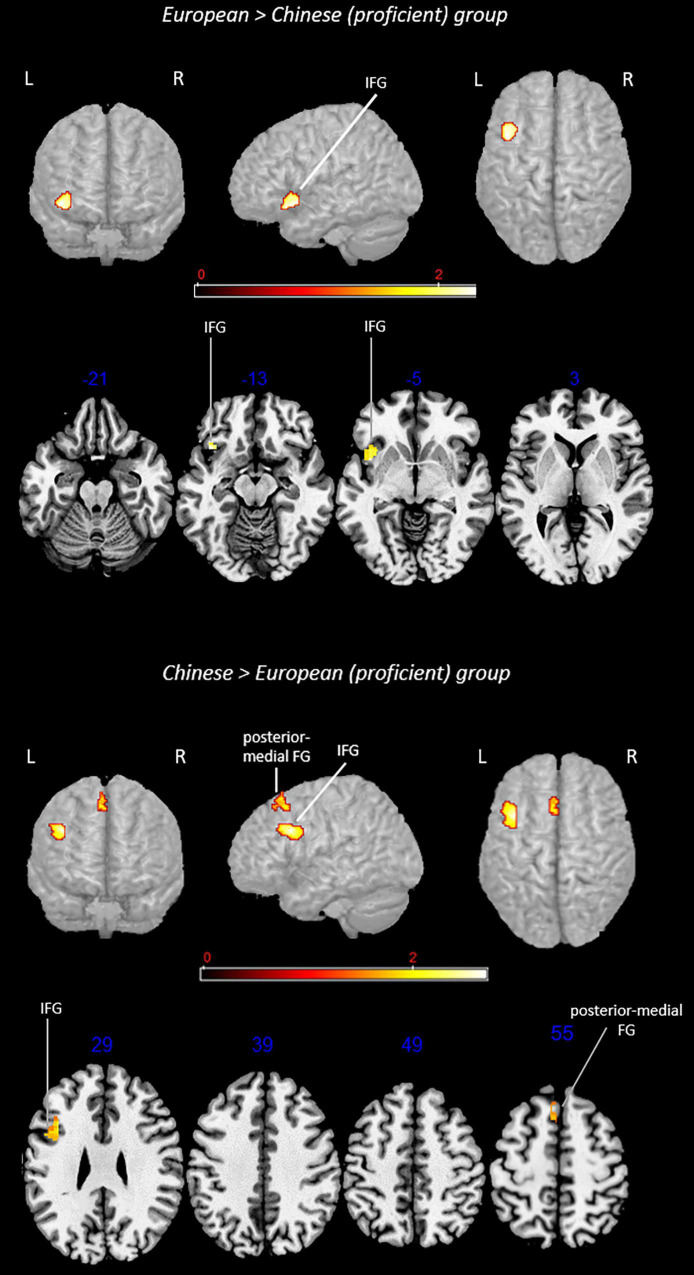
Rendered group-specific functional activations associated with L2 (i.e., English) in proficient bilinguals. Rendered anatomical depiction (in neurological convention) of contrast analysis results specifically associated with proficient L2 in the European and Chinese groups. IFG, inferior frontal gyrus; L, left hemisphere; posterior-medial FG, posterior-medial frontal gyrus; R, right hemisphere. On axial slices, numbers in blue indicate *z*-coordinates in MNI space. Bars indicate z-scores.

The main effect and contrast analysis results are detailed in Table 3. Figure 6 shows the results from main effect analyses and Figure 7 from contrast analyses.

#### Main Effect: L2 in the Proficient European Group

This analysis included 12 experiments, 140 subjects, and 113 foci. L2-associated activation clusters were found in the left (i) superior frontal gyrus and (ii) insula.

#### Main Effect: L2 in the Proficient Chinese Group

This analysis included 11 experiments, 119 subjects, and 54 foci. In this group, L2-associated activations were found in the left (i) inferior frontal gyrus (including regions associable with DLPFC) and (ii) posterior-medial frontal gyrus.

Contrast analyses included 33 experiments, 259 subjects, and 167 foci.

#### Conjunction Analysis: Proficient L2 in the European Group ∩ Proficient L2 in the Chinese Group

The analysis did not provide any suprathreshold clusters.

#### Subtraction Analysis: Proficient L2 in the European Group > Proficient L2 in the Chinese Group

This comparison showed activations in the left (i) inferior frontal gyrus.

#### Subtraction Analysis: Proficient L2 in the Chinese Group > Proficient L2 in the European Group

This comparison provided activations in the left (i) inferior frontal gyrus and (ii) posterior-medial frontal gyrus.

## Discussion

It is widely known that second language (L2) generally poses greater cognitive demands to bilinguals than the first language (L1), resulting in the recruitment of wider and/or additional brain regions (see meta-analyses by Indefrey, 2006; Sebastian et al., 2012; Liu and Cao, 2016; Cargnelutti et al., 2019). Nevertheless, the impact of linguistic distance is not completely clear. We identified two groups, both having English as L2, and explored whether the L2 functional network differed between bilinguals having as L1 either an Indo-European language structurally close to English or Chinese, which belongs to the Sino-Tibetan family and is structurally distant to English (Chiswick and Miller, 2005; Chang et al., 2015). We also controlled for the effects of L2 AoA and proficiency. Although, in these analyses, we could only control for one factor at a time (therefore, the group of late bilinguals included both low- and high-proficiency bilinguals and the group of proficient bilinguals included both early and late bilinguals), we observed that the resulting suprathreshold clusters were independent of the other factor (e.g., clusters in the group of late bilinguals were driven by both low- and high-proficiency bilinguals), indicating that AoA and proficiency are likely to shape independently—at least partially—the functional network associated with L2. Findings from the current meta-analysis provide a glimpse of the mechanisms that regulate brain response to L2 with important implications for research and, possibly, clinical work.

### Modulatory Effect of Linguistic Distance

The first analysis included all the selected studies independently of language domain, task, and presentation modality or factors relevant to bilingualism, such as AoA or proficiency. Interestingly, contrast analysis revealed a specific activation of the left insula for the European group and of the inferior frontal gyrus in the territory of the DLPFC for the Chinese group. Furthermore, the Chinese group selectively activated a cluster peaking in the intraparietal sulcus and extending to both IPL and SPL.

Activation of the insula and of the DLPFC was not surprising as these areas subserve more general higher cognitive functions including cognitive control, monitoring, and attention. Although not language-specific, these functions were observed to play a relevant role in language, particularly in cognitively demanding situations such as those posed by bilingualism. Concerning the specific activation in the European group, the insula has a relevant role in diverse cognitive functions including language (Oh et al., 2014) and is particularly involved in supporting the execution of tasks posing conflict and interference (Wager et al., 2005). In light of these findings, it could appear counterintuitive for this region to be specifically recruited in bilinguals knowing closer languages. In point of fact, one may hypothesize a greater need for cognitive support when dealing with two languages significantly differing in many respects (e.g., phonology and writing system) due to the need to switch to a different processing modality (e.g., the way graphemes are converted into phonemes).

Nevertheless, there is strong evidence that handling two languages is more challenging when they share linguistic features rather than when they are more structurally distant (Paradis, 1994, 2004, 2009; Fabbro, 1999); this is also shown by interference occurring, in specific conditions, between cognate words (Colomé and Miozzo, 2010; Muscalu and Smiley, 2019). This phenomenon was also described in some clinical studies in which languages sharing cognate words were observed to interfere with each other, thus hampering recovery in some bilingual patients with aphasia (Kurland and Falcon, 2011).

Regarding the Chinese group, selective activation of another crucial area involved in general cognitive functions was observed, namely, the DLPFC (MacDonald et al., 2000; Mansouri et al., 2007). In bilinguals, the DLPFC located in the inferior frontal gyrus included the control network, which regulates selection and control, in particular when a weaker language is in use (Abutalebi and Green, 2007, 2016). A cluster in the inferior/middle frontal gyrus (MNI coordinates: *x* = -44, *y* = 18, and *z* = 20) was also found in the meta-analysis by Liu and Cao (2016) with an L2 which was orthographically deeper (i.e., opaquer) than L1, as in the case of Chinese vs. English. The authors hypothesized that this area was recruited to handle the greater difficulty to coordinate sounds and meaning when L2 is an opaque language.

Taken together, these findings seem to suggest that, irrespective of similarity between known languages, bilinguals do tend to strongly rely on cognitive resources when dealing with their L2; however, the cognitive demands posed by closer vs. more distant languages could be different and translate into the specific recruitment of different areas. This hypothesis is more deeply discussed in the next paragraphs, where we illustrated the results obtained when controlling for AoA and proficiency.

The other specific activation for the Chinese group involved the IPL and SPL.^2^ The IPL has been mainly (although not uniquely) associated with phonological processing in both Chinese (Wu et al., 2012) and European languages (Hartwigsen et al., 2010) as well as with lexical knowledge in both (Mechelli et al., 2004; Abutalebi et al., 2015). Inspection of the contrasts contributing to this cluster showed that they pertained to different language domains and suggested that this activation was not task-specific.

The cluster we found also covered the SPL, which is generally recruited during spelling-to-sound mapping (Taylor et al., 2013) and acts as a station for audio-visual multisensory integration (Molholm et al., 2006). This area was observed to be particularly crucial for Chinese language processing, for instance when processing characters across diverse tasks (e.g., orthographic and semantic; see meta-analysis by Wu et al., 2012), but it is also involved in processing Japanese Kanji characters (Matsuo et al., 2000). For this reason, the SPL is considered a typical logographic language area, with a hypothesized involvement in holistic visuospatial processing (Bolger et al., 2005).

Activation of this area can be tentatively explained by drawing on the assimilation-accommodation hypothesis (Perfetti et al., 2007), which postulates that bilinguals may either rely on assimilation (i.e., applying the same procedures developed for L1 to L2 as well) or on accommodation (i.e., “abandoning” the procedures associated with L1 processing to activate those specific for L2). Our results cautiously suggest that Chinese-English bilinguals tend to rely on assimilation when using English.

Overall, these findings (Liu and Cao, 2016) suggest that the application of long-established mechanisms, although not perfectly fitting with the new language demands, could be the default strategy used to deal with a new distant language. This can more likely occur with late language learning, as findings the related paragraph seem to suggest. Bilinguals would use these mechanisms as long as these are effective and develop new-language-specific processing mechanisms only when those associated with L1 are inefficient.

Another interesting activation cluster in the Chinese—but not the European—group was represented by the posterior-medial frontal cortex, although it did not survive the statistical thresholds in the subtraction analysis. The cluster was located in close proximity to the pre-SMA/ACC region described by Abutalebi and Green, another fundamental station in language control, as it is involved in appropriate language selection and switching between languages (Abutalebi and Green, 2007, 2016). This activation, too, showed the high cognitive demand posed by L2 in this group of bilinguals.

These preliminary results probably reflect the activation clusters that are more tightly associated with the two conditions (closer vs. more distant language pairs). These activations are also likely to bypass language domain-specificity and represent the brain areas that are generally recruited when performing any task in L2, as they can reflect the cognitive load of managing an additional language (see also the exploratory lexical-semantic analysis in Supplementary Results).

### Age of Appropriation Effect

Several previous meta-analyses addressed the role of AoA and showed that, overall, late language learning (typically after the age of 6 years) is associated with the recruitment of additional and/or wider brain areas (Liu and Cao, 2016; Cargnelutti et al., 2019). This reflected a higher cognitive effort with respect to early L2 acquisition. The impact of AoA can be even more interesting in relation to linguistic distance: one may hypothesize that learning a new structurally distant language might be more demanding when it takes place late, therefore, when the cognitive mechanisms for L1 are already established.

For this reason, we replicated the analysis while controlling for AoA. Unfortunately, data for early bilinguals were limited, and therefore, comparison between early and late bilinguals was not possible. However, we can make indirect inferences on the results of late bilinguals. Our analyses showed that the European group activated specifically the left insula and hippocampus, whereas the Chinese group activated the left DLPFC and SPL. The main effect analysis showed that this group also activated the posterior-medial frontal gyrus. Also, in this case, the two groups did not share any suprathreshold activation clusters.

Findings for the Chinese group replicate those achieved for the whole group (i.e., without distinction based on AoA), suggesting that the specific activations we observed could be more relevant for the Chinese bilinguals having learned English late. This is reasonable as greater cognitive control—reflected in the specific activation of the DLPFC—can be necessary to learn L2 when the L1 network is already developed. Furthermore, as previously commented, late learners may tend to approach a new language by first applying—and, if successful, keeping—the same cognitive mechanisms developed to process L1 (assimilation). Activation of the SPL in late bilinguals suggested that this could be the case.

Concerning the European group, it was interesting to observe that the left insula was activated for the whole group. By inspecting the contrasts contributing to the cluster, we observed that these were represented by tasks involving potential conflict, for instance, lexical decision (i.e., decide if letter strings were real words or not). This finding contributes to supporting the previously mentioned hypothesis of insula recruitment to solve potential conflicts, which are amplified when dealing with L2.

With regard to the other European-group-specific activation, this did not match any macroanatomic probability map but it predominantly included the hippocampus. Contrasts contributing to this cluster mostly emerged from the comparison of morphological tasks in late vs. early bilinguals, suggesting that the hippocampus might be more likely recruited in the case of late AoA. This specific activation was observed to be driven by tasks tapping grammar. This was in agreement with the memory models describing language learning vs. acquisition (Paradis, 1994, 2004, 2009; Ullman, 2001, 2005, 2006). In fact, late learning involves the explicit memory system, whereas early acquisition relies on implicit processes, allowing the automatic performance of these tasks. In light of this theoretical background, the European group could rely on hippocampal activation, which is fundamental for memory retrieval, when performing morphological tasks in English. The different European languages included in our study are significantly more similar to English than Chinese (Chiswick and Miller, 2005). Accordingly, it is plausible that European bilinguals mainly rely on direct recall to perform these tasks in order to bypass potential interference between grammar rules of close languages. A retrieval could be a more effective strategy, whereas recruitment of regions supporting cognitive processing might be more successful for Chinese bilinguals. Another hypothesis comes from the recently observed role of the hippocampus in the flexible use and processing of language (Covington and Duff, 2016) and, remarkably, in associative learning (Brasted et al., 2003). Accordingly, its activation could reflect a more efficient learning process, where the potential obstacle represented by late AoA is overcome by the development of flexible and associative strategies for new rule application. These are only speculations, which need to be confirmed by a sufficient number of studies comparing activations associated with the same linguistic tasks in different groups of bilinguals; in this way, it would be possible to understand whether hippocampal involvement depends on specific tasks, languages, or profile of bilinguals (e.g., AoA and proficiency).

### Proficiency Effect

Another crucial aspect concerns the role of proficiency as discussed in previous meta-analyses (Sebastian et al., 2012; Cargnelutti et al., 2019): low proficiency is associated with greater cognitive effort and then with greater activation of areas involved in cognitive control. The role of proficiency in relation to linguistic distance could be particularly interesting to explore.

As previously detailed, we could not make a direct comparison between low- and high-proficiency bilinguals owing to the paucity of available data. The exploratory analysis carried out on bilinguals who were proficient in their L2 shows—partially unexpectedly—that both the European and Chinese groups activated regions supporting general cognitive functions. European bilinguals activated the insula bilaterally, although this activation emerged from the main effect analysis only and not from the subtraction analysis (again, probably due to the paucity of studies, which is a limitation especially in subtraction analyses). In contrast, the Chinese group activated specifically the posterior-medial frontal gyrus. Taken together, these findings indicate the role of these regions in supporting even proficient performance.

The insula was previously observed to activate in balanced bilinguals (i.e., bilinguals with native-like performance in both languages) compared with less proficient bilinguals and also in response to increased task difficulty (Chee et al., 2004). This seems to confirm that this region is recruited to cope with increased cognitive demands (Vigneau et al., 2011). In light of these findings, we can also hypothesize that the insula is activated when L2 is mastered very well: a very good mastery of more than one language is a demanding process but the insula could promote an anyway proficient performance.

By looking at peak location, it was similar between proficient bilinguals and late bilinguals (note that analysis in the group of late bilinguals included six contrasts referring to high-proficiency bilinguals and 10 to low-proficiency bilinguals, suggesting that a similar result could have been affected independently by both AoA and proficiency). For both groups, we can hypothesize a similar role of the insula in supporting the cognitive effort, although for different reasons: (i) the need for proficient bilinguals to perfectly master the two languages in conflict situations (i.e., bilingual environment) and (ii) the need for late bilinguals to overcome obstacles posed by late learning. Therefore, the other activation clusters are likely to work in synergy with the insula to meet the specific demands of the two conditions.

Regarding the Chinese group, the lack of SPL activation in this analysis is noteworthy. Although a direct comparison between high- and low-proficiency bilinguals was not possible, we could speculate that a high level of proficiency can be more easily reached when accommodation—rather than assimilation—processes take place and may also be prompted by accommodation. High proficiency is more likely gained throughout proper recruitment of the areas supporting general cognitive functions than by areas specifically associated with a given language.

## Conclusion

This meta-analysis showed recurrent L2-associated activation of regions involved in general higher cognitive functions, despite some differences between the two groups of bilinguals. These differences probably reflected the different cognitive efforts—therefore, recruitment of different cognitive resources—associated with L2 depending on a different degree of linguistic distance with L1.

The insula appeared to be mainly activated to solve potential conflicts between structurally closer languages, whereas the DLPFC and posterior-medial frontal gyrus (pre-SMA/ACC) when the two languages differ to a greater extent. The crucial SPL activation in the Chinese group seems to support a general tendency for reliance on assimilation when processing English, but this was not likely to be the case for proficient bilinguals.

However, we highlighted the limitations of this study and declared its almost exploratory intent. These limitations were not completed due to the study design. First, a small number of studies met the inclusion criteria, which was probably the reason why we observed fewer-than-expected functional activations. Another limitation concerned the characteristics of the bilingual populations, for instance, the small number of early Chinese-English bilinguals. Furthermore, proficiency was not assessed in an objective way in all the included studies, preventing a reliable classification based on this criterion. Therefore, we suggested a more accurate investigation of this variable in future studies, possibly evaluating the degree of language exposure as well. Another limitation is the lack of a robust definition of linguistic distance and the fact that this was not assessed and quantified in neuroimaging studies on bilinguals.

Nevertheless, these preliminary findings could help us better understand how linguistic distance interacts with other relevant factors such as AoA and proficiency in defining the L2 functional network. Our findings may also provide some useful hints from a clinical viewpoint: knowing which brain regions are specifically involved in language processing in these bilinguals may contribute to understanding the impact of potential damage on L1 and L2 performance. This study stresses the importance of cognitive control regions and suggests including specific training of these abilities in the rehabilitation of patients developing bilingual aphasia.

## Data Availability Statement

The datasets presented in this article are not readily available because the authors are still implementing it to conduct further meta-analyses. Requests to access the datasets should be directed to corresponding author.

## Author Contributions

EC: manuscript search, manuscript scrutiny, analysis, writing – original draft, and writing – review and editing. BT and FF: manuscript scrutiny, supervision, and writing – review and editing. All authors contributed to the article and approved the submitted version.

## Conflict of Interest

The authors declare that the research was conducted in the absence of any commercial or financial relationships that could be construed as a potential conflict of interest.

## Publisher’s Note

All claims expressed in this article are solely those of the authors and do not necessarily represent those of their affiliated organizations, or those of the publisher, the editors and the reviewers. Any product that may be evaluated in this article, or claim that may be made by its manufacturer, is not guaranteed or endorsed by the publisher.
