# Supplementary material for: Effects of Linguistic Distance on Second Language Brain Activations in Bilinguals: An Exploratory Coordinate-Based Meta-Analysis
Source: Front Hum Neurosci. 2022 Jan 6;15:744489. doi: 10.3389/fnhum.2021.744489 (PMC8770833; doi:10.3389/fnhum.2021.744489)
Supplement: Supplementary file 1 [file Data_Sheet_1.pdf]

SUPPLEMENTARY RESULTS

L1 in the European group

SUPPLEMETARY TABLE S1. ALE meta-analysis results for L1: main effects and contrasts between L1 and L2 in the European group.

Cluster (local maxima)		MNI coordinates x	y	z	Cluster size (voxels)	*z*-score
**L1** (18 experiments, 208 subjects, and 136 foci)						
1	L precentral gyrus	-50	2	40	122	4.36
	L precentral gyrus	-52	0	48		
2	L inferior frontal gyrus (BA 44)	-56	10	12	296	5.20
	L temporal pole (area TE3)	-60	6	-2		
**L1 ∩ L2** (48 experiments, 576 subjects, and 401 foci)						
1	L postcentral gyrus	-48	-6	38	15	-
**L1 > L2** (48 experiments, 576 subjects, and 401 foci)						
1	L precentral gyrus	-54	-2	44	23	1.93
2	L rolandic operculum (area TE3)	-58	4	2	80	2.26
**L2 > L1** (48 experiments, 576 subjects, and 401 foci)						
1	L inferior frontal gyrus	-42	16	-12	111	2.33

Anatomical localization, macroanatomic area and, when provided, cytoarchitectonic location (in parentheses) are indicated.

L1 in the Chinese group

SUPPLEMETARY TABLE S2. ALE meta-analysis results for L1: main effects and contrasts between L1 and L2 in the Chinese group.

Cluster (local maxima)		MNI coordinates x	y	z	Cluster size (voxels)	*z*-score
**L1** (20 experiments, 265 subjects, and 160 foci)						
1	L inferior frontal gyrus	-46	22	28	393	5.01
**L1 ∩ L2** (44 experiments, 585 subjects, and 333 foci)						
1	L inferior frontal gyrus (BA 44)	-54	12	26	95	-
**L1 > L2** (44 experiments, 585 subjects, and 333 foci)						
1	L inferior frontal gyrus	-42	24	16	88	2.43
**L2 > L1** (44 experiments, 585 subjects, and 333 foci)						
1	L precentral gyrus	-58	6	26	77	2.18

Anatomical localization, macroanatomic area and, when provided, cytoarchitectonic location (in parentheses) are indicated.

L2 lexical-semantics

We explored functional activations specifically associated with lexical-semantics (at word but not sentence level), which represented the most studied language domain in our paper sample. Main effect analysis included 10 experiments, 130 subjects, and 70 foci for the European group and 18 experiments, 207 subjects, and 124 foci for the Chinese group.

We observed the specific activation of the right insula for the European group and of the left superior parietal lobule (SPL) for the Chinese group (for the right insula role in executive control associated with L2 lexical-semantics, see Sulpizio et al., 2020; for involvement of SPL in several Chinese language tasks, see Wu et al., 2012).

SUPPLEMETARY TABLE S3. ALE meta-analysis results for L2 (i.e., English) lexical-semantics: main effects and contrasts in the European and Chinese groups.

Cluster (local maxima)		MNI coordinates x	y	z	Cluster size (voxels)	*z*-score
**European group** (10 experiments, 130 subjects, and 70 foci						
1	R insula	38	10	0	101	4.59
2	L N/A	-52	34	-18	231	4.55
**Chinese group** (18 experiments, 207 subjects, and 124 foci)						
1	L superior parietal lobule	-24	-74	42	141	4.33
2	L precentral gyrus	-56	8	28	89	4.28
3	L superior medial gyrus	0	14	50	103	3.57
**European group ∩ Chinese group** (28 experiments, 337 subjects, and 194 foci)						
No suprathreshold clusters						
**European group > Chinese group** (28 experiments, 337 subjects, and 194 foci)						
1	R insula	38	10	0	100	2.34
2	L inferior frontal gyrus	-36	20	-14	127	2.51
3	L N/A	-52	34	-18	45	1.90
**Chinese group > European group** (28 experiments, 337 subjects, and 194 foci)						
1	L superior parietal lobule	-26	-70	52	11	1.77

Anatomical localization, macroanatomic area and, when provided, cytoarchitectonic location (in parentheses) are indicated.
